# Viral Coinfection is Associated with Improved Outcomes in Emergency Department Patients with SARS-CoV-2

**DOI:** 10.5811/westjem.2021.8.53590

**Published:** 2021-10-26

**Authors:** Elizabeth M. Goldberg, Kohei Hasegawa, Alexis Lawrence, Jeffrey A. Kline, Carlos A. Camargo

**Affiliations:** * Brown University, Department of Emergency Medicine, Providence, Rhode Island; † Massachusetts General Hospital/Harvard Medical School, Department of Emergency Medicine, Boston, Massachusetts; ‡ Wayne State University School of Medicine, Department of Emergency Medicine, Detroit, Michigan

## Abstract

**Introduction:**

Coinfection with severe acute respiratory syndrome-coronavirus 2 (SARS-CoV-2) and another virus may influence the clinical trajectory of emergency department (ED) patients. However, little empirical data exists on the clinical outcomes of coinfection with SARS-CoV-2

**Methods:**

In this retrospective cohort analysis, we included adults presenting to the ED with confirmed, symptomatic coronavirus 2019 who also underwent testing for additional viral pathogens within 24 hours. To investigate the association between coinfection status with each of the outcomes, we performed logistic regression.

**Results:**

Of 6,913 ED patients, 5.7% had coinfection. Coinfected individuals were less likely to experience index visit or 30-day hospitalization (odds ratio [OR] 0.57; 95% confidence interval [CI], 0.36–0.90 and OR 0.39; 95% CI, 0.25–0.62, respectively).

**Conclusion:**

Coinfection is relatively uncommon in symptomatic ED patients with SARS-CoV-2 and the clinical short- and long-term outcomes are more favorable in coinfected individuals.

## INTRODUCTION

According to the US Centers for Disease Control and Prevention, as of October 2021 the severe acute respiratory syndrome coronavirus 2 (SARS-CoV-2) virus has caused an estimated 684 hospitalizations per 100,000 population and 711,020 deaths in the United States.^1^ Emergency clinicians decide which patients with coronavirus 2019 (COVID-19) to admit to the hospital and these decisions typically take into account patient age, need for supplemental oxygen, and other clinical and laboratory metrics, as well as anticipated clinical trajectory.^2^,^3^ Coinfection with SARS-CoV-2 and another virus may influence the short- and long-term clinical outcomes, and thus co-infection status could inform clinical decision-making in the emergency department (ED). However, little empirical data exists on the clinical outcomes of coinfection with SARS-CoV-2.

With the introduction of reverse transcription real-time polymerase chain reaction assays the detection of viral coinfections has grown.^4^ However, interpretation of these test is challenging as studies with short- and long-term clinical outcomes are scant, particularly for SARS-CoV-2 coinfection. Some reports of viral coinfection preceding the COVID-19 pandemic suggest higher disease severity with coinfection,^5^ while others report no relationship between multiple (non-SARS-CoV-2) respiratory viral infections and disease severity.^6^ The rate of coinfection and its potential impact on clinical outcomes likely depends on the particular virus, the means of detection, the patient’s demographics, and location of the study.^7^ Therefore, an evaluation of coinfection rates and outcomes for SARS-CoV-2 is necessary and important.

A cross-sectional study of 1206 patients with respiratory symptoms revealed that 20.7% were positive for SARS-CoV-2 and at least one additional virus.^8^ The most common coinfections were rhinovirus/enterovirus (6.9% of 116 specimens), respiratory syncytial virus (RSV) (5.2%), and non-SARS-CoV-2 coronaviruses (4.3%), but results were limited to a three-week period in March 2020 in a single region. Likewise, a meta-analysis (total of 1800 subjects)^9^ reported a 11.7% coinfection rate; however, because serum antibody studies that indicate both recent and acute infection were included, this may have artificially increased the coinfection rate. Neither study addressed clinical outcomes in coinfected patients.

Therefore, our objectives were to determine the frequency of SARS-CoV-2 and any additional respiratory virus (coinfection) among ED patients. Secondarily, we were interested in comparing encounters with and without coinfection in terms of the following: a) baseline characteristics; b) short-term outcomes (hospitaliza-tion at the index ED visit); and c) 30-day clinical outcomes (hospitalization within 30 days of index ED visit; severe COVID-19 within 30 days defined as intubation with mechanical ventilation and/or death). We hypothesized that patients with SARS-CoV-2 coinfection would be more likely to experience unfavorable short- and long-term outcomes.

## METHODS

### Data Source

The national Registry of Suspected COVID-19 in Emergency Care (RECOVER) network recorded clinical data on 35,120 ED patient encounters for COVID-19 symptoms.^10^ Encounters occurred between the first week of February 2020 and the fifth week of October 2020. Of the sites contributing to the registry 60% were community hospitals without a residency program.^10^ Clinical characteristics and outcomes were extracted from electronic health records by automated download and then supplemented by medical record review by trained research personnel. Best practices in medical record review studies were adhered to,^11^ including the following: abstractor training; case selection criteria; variable definition; abstraction forms; performance monitoring; institutional review board approval; and data management plan.

### Study Design

In this retrospective cohort analysis, we included RECOVER network encounters by adults **≥** 18 years from 86 hospitals in 27 states. Eligibility for enrollment required that a molecular diagnostic test to have been performed in the ED setting or within 24 hours for patients with possible SARS-CoV-2 infection. Registry guidance advised that patients without suspected infection but who had swab testing performed in the ED only to comply with a hospital screening policy for admissions or preoperative testing be excluded.^12^ In total, 204 defined questions were asked about encounters falling in seven domains: 1) visit information; 2) demo-graphics, symptoms and risk factors; 3) vital signs; 4) past medical history; 5) current medications; 6) test results; and 7) outcomes. The registry collected 47 questions about test results including whether extended viral testing was performed and the results of that testing. No effort was made to standardize the type of viral testing performed for each person or by site; however, only patients with molecular testing for SARS-CoV-2 were eligible for inclusion. The criterion standard for SARS-CoV-2 diagnosis required a positive molecular test (as opposed to antigen testing) from a swabbed sample from the nasopharynx. Coinfections were detected by molecular testing of separate swabs taken simultaneously. The local hospital institutional review board (IRB) approved the study (IRB # 1586472-1), and informed consent was waived for this minimal risk study.

**Population Health Research Capsule**

What do we already know about this issue?

*Some reports of viral coinfection preceding the COVID-19 pandemic suggest higher disease severity with coinfection.*

What was the research question?

*What is the rate of SARS-CoV-2 coinfection among emergency department patients and its impact on clinical outcomes?*

What was the major finding of the study?

*Of 6,913 ED patients, 5.7% had coinfection. Coinfected individuals were less likely to experience index visit or 30-day hospitalization.*

How does this improve population health?

*Adults with coinfection with SARS-CoV-2 do not have worse clinical outcomes, compared to those without coinfection. This suggests that the impact of extended viral panels on clinical management is limited.*

### Exposure

The primary exposure was coinfection by any respiratory virus(es) (eg, adenovirus, endemic coronavirus, influenza virus) at the index ED visit.^10^ Thus, we excluded encounters that did not report results of other viral testing.

### Outcomes

The outcomes of interest were hospitalization at the index ED visit, any hospitalization within 30 days of index ED visit, and severe COVID-19. Severe COVID-19 was defined as intubation with mechanical ventilation and/or death within 30 days.^10^

### Statistical Analyses

We described the baseline characteristics and clinical presentation at the index ED visit as well as outcomes. To investigate the association between coinfection status with each of the outcomes, we then constructed unadjusted and adjusted logistic regression models. In the multivariable model, we adjusted for 10 potential confounders based on a priori knowledge: age; gender; race/ethnicity; hypertension; cardiovascular diseases; chronic obstructive pulmonary disease; other chronic lung diseases; obesity; diabetes; and cancer.^13^ We performed the analysis using R version 4.0.1 (R Foundation for Statistical Computing, Vienna, Austria).

## RESULTS

**Figure 1:**
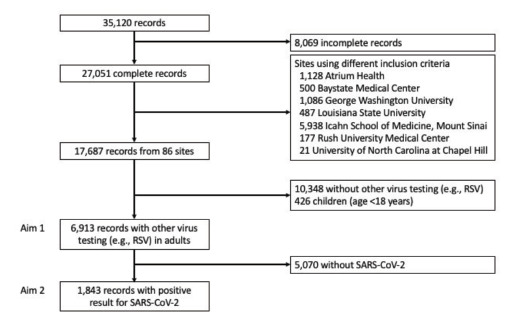
Inclusion flow diagram. Among 35,120 records, 27,051 were complete and 17,687 used the same inclusion criteria. Of these, 6,913 records contained data on non-SARS-CoV-2 virus testing. We used these records to determine the frequency of SARS-CoV-2 and any additional respiratory virus (coinfection) among ED patients (Aim 1). We then used all records remaining that had positive SARS-CoV-2 results for Aim 2, where we compared encounters with and without coinfection in terms of a) baseline characteristics; b) short-term outcomes (hospitalization at the index ED visit), and c) 30-day clinical outcomes (hospitalization within 30 days of index ED visit; severe COVID-19 within 30 days defined as intubation with mechanical ventilation and/or death). *SARS-CoV-2*, severe acute respiratory syndrome coronavirus 2; *ED*, emergency department.

After exclusion of records from seven sites that used different inclusion criteria (n = 9,364), incomplete records (n = 8,069), children (n = 426), and encounters without non-SARS-CoV-2 viral testing (n = 10,348), our analytic sample included 6913 patient encounters (Figure 1). Among these 6,913 encounters, the median age was 59 (interquartile range 46–71) years and 49% were female.

Overall, 1,843 (27%) patients had SARS-CoV-2 of whom 1,726 (94%) had SARS-CoV-2 alone and 117 (6%) were coinfected with an additional virus (Table 1). Those with coinfection were younger and more likely to be non-Hispanic Black. Additionally, there were significant differences by coinfection status for heart rate and oxygen saturation on presentation, with patients with coinfection having higher heart rates and oxygen saturation on room air. The most common additional viruses were RSV (60/117, 51%), rhinovirus (20, 17%), and non-SARS-CoV-2 coronaviruses (15, 13%).

Encounters in which patients were coinfected were significantly less likely to result in hospitalization at the index ED visit (51% vs 68%, *P*<0.001), and less likely to result in any hospitalization within 30 days (55% vs 76%, *P*<0.001), compared to encounters with patients testing positive for SARS-CoV-2 alone. In the multivariable model, compared with patients who had only SARS-CoV-2, those with SARS-CoV-2 and at least one additional virus had lowered adjusted odds of hospitalization at the index ED visit (odds ratio [OR] 0.57; 95% confidence interval [CI], 0.36–0.90) and hospitalization within 30 days (OR 0.39; 95% CI, 0.25–0.62). Coinfected patients did not have an increased odds of severe COVID-19 (OR 0.76; 95% CI, 0.46–1.24).

## DISCUSSION

In this retrospective cohort study, which included 86 EDs, we found that coinfection occurs infrequently (5.7%) among symptomatic ED patients, and coinfection was not associated with hospitalization or other unfavorable short- and long-term outcomes. To our knowledge this is the first study examining clinical outcomes of symptomatic ED patients with SARS-CoV-2 based on coinfection status.

There are several potential explanations for why coinfection appeared to have a “protective” effect. First, ED patients found to be coinfected could have had an asymptomatic SARS-CoV-2 infection and may have had presenting symptoms from their other virus. Another explanation could be that individuals with high rates of prior viral exposure – through their occupation or social behaviors – may have primed their immune system with other coronaviruses and respiratory pathogens and may, therefore, have experienced less severe COVID-19.^14^ Non SARS-CoV-2 (endemic) coronaviruses share sequence homology with SARS-CoV-2, and immune responses can cross-react with SARS-CoV-2 antigens, eg, through long-lasting memory T cells.^15^,^16^ Finally, SARS-CoV-2 could have been attenuated by other viruses (viral interference), or other viruses could have been the primary infection and could have initiated a partially helpful immune response reducing the severity of SARS-CoV-2 illness.^15^ These potential explanations merit further study.

Compared to other published studies on coinfection, we found similar rates of other viruses, with RSV being most common.^8^,^9^,^17^ RSV, while most recognized as the causative agent of infant bronchiolitis, causes severe infection in older adults with a morbidity and mortality similar to influenza.^18^ Symptoms of RSV are similar to COVID-19, but nasal congestion and wheezing are typical.^18^ Age-related immune senescence, whereby older adults may have lower protective serum antibodies against viral pathogens, increases vulnerability of this population to respiratory infection. Rhinovirus is an important cause of illness in school-age children, causing sputum production, myalgias, and nasal congestion, but may be less serious in adults. Similarly, endemic coronaviruses typically cause mild nasal congestion, dyspnea, and sputum production,^18^ and rarely lead to hospitalization. While our study focused on viral coinfections, a recent study of 8649 inpatients in the United Kingdom examined bacterial coinfections in patients admitted to the hospital with COVID-19 and found that bacterial coinfections are rare, most are secondary (occurring more than two days after hospital admission), and are not associated with inpatient mortality. The UK study concluded that empirical antimicrobial prescribing should be restricted.^19^

Clinical and policy implications of our study include that viral coinfection status does not confer greater risk of clinical deterioration among ED patients. Based on our results adults with multiple viral pathogens (coinfection with SARS-CoV-2) do not have worse clinical outcomes, compared to those without infection, which suggest that the impact of extended viral panels on clinical management is limited. A study by Burk et al^20^ found that coexisting viral and bacterial pathogens conferred greater mortality in community-acquired pneumonia (OR 2.1, 95% CI, 35.1–53.3%). However, our data shows this not to be true for coinfection with another virus in COVID-19. Extended respiratory panels are costly at $3,450^21^ per specimen and may not be advised unless needed for inpatient cohorting (keeping patients with similar pathogens in the same room), antiviral treatment purposes (eg, oseltamivir in early influenza illness), public health surveillance, or for special populations. In observational studies, however, patients with positive influenza results receive fewer antibiotics, undergo fewer diagnostic tests, and are less likely to be hospitalized; thus, extended panels may have utility in patients requiring hospitalization.^22^ It should be noted that the “twin-demic” of influenza and COVID-19 did not occur this year, likely due to high vaccination rates against flu and protective measures such as distancing and mask wearing. Without these protective measures we may have seen greater coinfection rates in our sample.

Multiple guideline groups have addressed the role of laboratory testing for viruses in different patient populations.^7^ Generally, testing may play a more important role in the management of severely ill patients and immunocompromised patients, but less so in relatively healthy adults and children. Guidelines suggest that hematology and oncology patients,^23^ transplant patients,^24^ intensive care unit patients,^25^ and pediatric patients with underlying disease^26^ are good candidates for extended viral pathogen testing. Additionally, testing is useful for public health investigations of emerging pathogens such as SARS-CoV-2, epidemiological investigations. and for infection control. A pragmatic approach should be taken in the ED where testing is considered when it may impact clinical decisions or support patient management. Clinical symptoms associated with different viruses causing respiratory illnesses overlap and are often indistinguishable from illness due to bacteria based on clinical symptoms alone. Clinicians should understand that multiple viruses can cause similar signs and symptoms and laboratorians should base testing algorithms on current circulating pathogens in their region and emerging infections in other regions of the world.

Future directions include evaluating coinfection status among patients who are asymptomatic. Most studies published on this topic include only symptomatic patients, and coinfection rates may be higher in this population.^27^ Presence and timing of outbreaks, such as influenza, can influence the other viral pathogens that are detected on samples, and further studies during different seasons and for different outbreaks would be useful. However, with increasing global travel. circulation patterns of viruses and dominant types can change from year to year.^28^

## LIMITATIONS

One potential limitation of this work is that coinfection rates may be lower than true rates, given clinician and site variability in respiratory virus testing. Additionally, as this was a retrospective analysis site investigators did not change clinical care or practice patterns. Thus, it was at the discretion of the emergency clinician whether to order an extended viral panel or solely a COVID-19 test. The ordering of extended viral panels is likely clinician, patient, and site specific. We also could not account for important confounders, such as smoking, frailty, and socioeconomic factors. Another possible limitation is that respiratory viruses are seasonal, and our data includes encounters from February–October 2020 only.

Sixty-seven percent of our included patients were admitted. This high rate of admission could suggest that extended viral panels were more often ordered on patients with higher disease severity. Thus, there is a potential issue of confounding by indication. Strengths of our study include its generalizability; our data represents ED encounters throughout the US. Although we have statistically significant inference with the sample size in our cohort, an external validation in a separate patient sample would further enhance generalizability of the inference. Patient presentations to the ED likely reflect those with clinically meaningful illness (vs serum antibody testing that was included in prior studies).^9^ Additionally, the RECOVER registry included a standardized data entry instrument and fidelity checks to enhance data quality.^10^

## CONCLUSION

We found that coinfection is relatively uncommon in patients with SARS-CoV-2 and the clinical short- and long-term outcomes for patients are more favorable in coinfected individuals. These findings provide insight into the clinical course of patients with coinfection and lend support to the theory that commonly encountered respiratory viruses could stimulate the immune response to protect individuals from SARS-CoV-2.^15^

## Figures and Tables

**Table 1:** Characteristics and clinical presentation of 1,843 adults with SARS-CoV-2 infection by coinfection status.

Variables	Any coinfection N = 117 (6%)	No coinfection N = 1,726 (94%)	P-value
Characteristics			
Age (yr), median, (IQR)	53 (41–66)	60 (46–71)	0.002
Female gender	54 (46)	843 (49)	0.64
Race/ethnicity			0.03
Non-Hispanic White	31 (26)	527 (31)	
Non-Hispanic Black	60 (51)	656 (38)	
Hispanic	14 (12)	263 (15)	
Other	12 (10)	280 (16)	
Smoking	14 (12)	161 (9)	0.44
Major comorbidities			
Hypertension	60 (51)	937 (54)	0.59
Ischemic heart disease	9 (8)	192 (11)	0.31
Heart failure	8 (7)	178 (10)	0.29
Asthma	22 (19)	211 (12)	0.054
COPD	12 (10)	174 (10)	0.99
Other chronic lung diseases*	4 (3)	44 (3)	0.79
Obesity	37 (33)	529 (31)	0.84
Diabetes	30 (26)	539 (31)	0.24
Hyperlipidemia	28 (24)	628 (37)	0.008
Cancer	13 (11)	189 (11)	0.99
HIV/AIDS	1 (1)	20 (1)	0.99
Organ transplantation	1 (1)	22 (1)	0.99
Alcohol abuse	3 (3)	104 (6)	0.18
Other substance use†	15 (13)	56 (3)	<0.001
ED presentation			
Heart rate (bpm), median (IQR)	98 (88–109)	95 (83–108)	0.03
Respiratory rate at presentation (per minute), median (IQR)	20 (18–21)	20 (18–22)	0.68
Oxygen saturation on room air (%), median (IQR)	97 (94–98)	95 (92–98)	<0.001
Respiratory virus testing			
Adenovirus	8 (7)	-	-
Endemic coronavirus	15 (13)	-	-
Human metapneumovirus	9 (8)	-	-
Influenza A	4 (4)	-	-
Influenza B	1 (1)	-	-
Influenza A & B	3 (3)	-	-
Parainfluenza viruses 1–4	6 (5)	-	-
RSV	60 (51)	-	-
Rhinovirus	20 (17)	-	-
Other viruses	14 (12)	-	-
Clinical outcomes			
Hospitalization at index ED visit	60 (51)	1,169 (68)	<0.001
High-flow oxygen	15 (13)	335 (19)	0.10
NIPPV	3 (3)	85 (5)	0.35
Intubation and mechanical ventilation	24 (21)	406 (24)	0.53
ECMO	1 (1)	55 (3)	0.25
ICU admission	28 (24)	499 (29)	0.28
Any hospitalization within 30 days‡	64 (55)	1,319 (76)	<0.001
Severe COVID-19§	25 (21)	498 (29)	0.10
Death	15 (13)	295 (17)	0.28

* Defined by pulmonary fibrosis, cystic fibrosis, bronchiectasis, or pulmonary hypertension

† Include cocaine, injection drugs, marijuana, methamphetamine, or opioid use

‡ Hospitalization immediately after the index ED visit or within 30 days from the index ED visit

§ Intubation with mechanical ventilation and/or death within 30 days from the index ED visit

*SARS-CoV-2*, severe acute respiratory syndrome coronavirus 2; *yr*, year; *IQR*, interquartile range; *COPD*, chronic obstructive pulmonary disease; *HIV/AIDS*, human immunodeficiency virus/acquired immunodeficiency syndrome; *BPM*, beats per minute; *COPD*, chronic obstructive pulmonary disease; *ECMO*, extracorporeal membrane oxygenation; *ED*, emergency department; *ICU*, intensive care unit; *NIPPV*, non-invasive positive pressure ventilation; *RSV*, respiratory syncytial virus.
